# Accelerating Cosmetic Innovation Through Next-Generation Computational Toxicology: From Structural Alerts to Molecular Dynamics and Artificial Intelligence for Skin Sensitization Assessment

**DOI:** 10.3390/toxics14090777

**Published:** 2026-08-31

**Authors:** Thomas Enrique Quintero-Trujillo, Liseth Diaz-Rojas, Helen Andrade, Paola Alfonso-Romero

**Affiliations:** Research, Development & Innovation, Belcorp, Tocancipa 251017, Colombia; handrade@belcorp.biz (H.A.); palfonso@belcorp.biz (P.A.-R.)

**Keywords:** skin sensitization, New Approach Methodologies (NAMs), computational toxicology, molecular dynamics, deep learning, artificial intelligence

## Abstract

Skin sensitization remains a critical toxicological endpoint in cosmetic ingredient development, particularly under accelerated innovation cycles and increasingly stringent safety requirements. Although NAMs have achieved substantial regulatory maturity for this endpoint, most computational approaches—including structural alerts, read-across, and quantitative structure–activity relationship models—rely predominantly on chemical structure and statistical associations, with limited representation of the molecular processes underlying sensitization. This perspective proposes a transparent and testable computational framework that integrates conventional chemical descriptors, reaction-domain information, covalent docking, molecular dynamics simulations, and machine learning methods. The framework is organized around the skin sensitization adverse outcome pathway and focuses on generating mechanistically informed descriptors associated with the molecular initiating event and selected molecular processes related to keratinocyte activation. These descriptors include reactive geometry, residue accessibility, interaction persistence, conformational behavior, and perturbation hypotheses involving the KEAP1–NRF2 regulatory axis. Rather than replacing established experimental NAMs or DAs, the proposed workflow is intended as a complementary, tiered evidence layer for the early prioritization of structurally characterized cosmetic ingredients and for guiding subsequent experimental testing. Its future value will depend on module-level validation, demonstration of incremental predictive performance, explicit applicability-domain and uncertainty assessment, computational scalability, and prospective comparison with established NAM outcomes.

## 1. Skin Sensitization as a Critical Endpoint in Cosmetic Product Development

The global cosmetics industry operates within increasingly rapid innovation cycles driven by evolving consumer preferences for novel product formats, technologies, and ingredients. However, this accelerated pace of innovation must be balanced with rigorous quality standards and compliance with current regulatory requirements, ensuring that consumer safety remains the highest priority.

Among the toxicological endpoints that critically influence product safety, skin sensitization has become particularly important in cosmetic product development. This relevance is reinforced by the growing proportion of consumers who perceive themselves as having sensitive skin, reaching as high as 71% in some populations [1]. Although self-perceived sensitive skin and allergic contact sensitization are clinically distinct conditions, the high consumer awareness of skin reactivity reinforces the demand for cosmetic products developed under increasingly stringent safety criteria. Consequently, there is increasing concern regarding the early identification of ingredients that may present skin sensitization risks. Furthermore, evidence reported by DeKoven et al. and Warshaw et al. [2,3,4,5] suggests an increasing prevalence of skin reactivity to a broader range of substances under controlled clinical patch-testing studies.

For this reason, assessing skin sensitization potential has become a routine component of cosmetic product development. Nevertheless, currently available experimental methods remain relatively time-consuming and costly and may present technical limitations when evaluating compounds with challenging physicochemical properties, such as highly insoluble or hydrophobic substances, strong redox-active chemicals, nanomaterials, or complex mixtures [6]. Delaying or deprioritizing these assessments may ultimately lead to late-stage product reformulations, development delays, increased development costs, and potential reputational risks for manufacturers.

## 2. Current Landscape of New Approach Methodologies for Skin Sensitization

Skin sensitization is one of the toxicological endpoints for which New Approach Methodologies (NAMs) have achieved the highest degree of regulatory maturity. Their development is grounded in the Adverse Outcome Pathway (AOP) framework for skin sensitization, which describes the mechanistic sequence leading from the covalent binding of electrophilic chemicals to skin proteins to the activation and expansion of antigen-specific T lymphocytes associated with allergic contact dermatitis. This mechanistic framework has enabled the progressive replacement of animal testing with complementary, mechanism-based alternative methods [7].

From a regulatory perspective, the most established NAMs address the first three key events (KEs) of the skin sensitization AOP. The Direct Peptide Reactivity Assay (DPRA) and related in chemico methods evaluate the chemical reactivity of test substances toward synthetic peptides containing cysteine (Cys) or lysine (Lys) residues, thereby representing the molecular initiating event of covalent protein binding [8]. KeratinoSens™ and related assays address keratinocyte activation, typically by measuring activation of the NRF2/ARE antioxidant response pathway [9]. Finally, several in vitro assays evaluate dendritic cell or monocyte activation, including h-CLAT, U-SENS™, IL-8 Luc, and GARD™skin, thereby covering the third key event of the AOP [10].

Despite these advances, no individual assay can reproduce the full biological complexity of the skin sensitization process. Consequently, one of the most significant developments in recent years has been the implementation of Defined Approaches (DAs), formalized in OECD Guideline 497. Rather than relying on a single assay, DAs combine information from multiple NAMs using fixed data interpretation procedures to improve predictive performance and increase confidence in hazard identification [11].

## 3. Computational Approaches for Skin Sensitization Assessment: Progress and Current Limitations

Within the fast-paced innovation cycles of the cosmetic industry, conventional experimental methods for assessing skin sensitization can become significant bottlenecks because of their cost, time requirements, and experimental complexity. Consequently, computational toxicology has emerged as an attractive strategy for supporting earlier and faster decision-making during product development. This evolution has reached a level of regulatory acceptance in which computational models such as SARA-ICE have been incorporated into OECD Guideline 497 as part of defined approaches for skin sensitization assessment [11].

Among the computational methodologies currently available, structural alerts and read-across remain two of the most widely adopted approaches. Structural alerts identify chemical reactive substructures that can undergo electrophilic reactions with nucleophilic residues in skin proteins, thereby representing the MIE of skin sensitization. In contrast, read-across predicts the toxicological properties of an unknown substance by extrapolating information from structurally similar compounds considered chemical analogues [12].

Despite their widespread application, both approaches present limitations detailed in Table 1 that may reduce confidence during early decision-making in cosmetic innovation.

Furthermore, skin sensitization is a highly complex biological process involving multiple interconnected key events within the AOP, including covalent protein binding, keratinocyte activation, dendritic cell maturation, and T-cell proliferation. Although QSAR and other machine learning models have demonstrated considerable predictive capability, they are primarily built upon molecular structural descriptors and statistical patterns extracted from existing experimental datasets. As a result, their ability to explicitly represent the biological mechanisms underlying skin sensitization remains inherently limited [20,24].

Consequently, these models generally capture only a subset of the biological events involved in skin sensitization, particularly those associated with the intrinsic chemical reactivity of the compound, while downstream cellular and immunological processes remain insufficiently represented. This limitation explains why QSAR models, despite their strong predictive performance, constitute only one component of the evidence integrated within Defined Approaches (DAs). Current regulatory strategies therefore rely on combining multiple complementary sources of evidence—including in chemico, in vitro, and, where available, in vivo data—to achieve broader coverage of the AOP key events and increase confidence in regulatory decision-making. To our knowledge, no previously published framework has integrated covalent docking, molecular dynamics simulations, AOP-oriented mechanistic descriptor generation and deep learning within a unified computational workflow explicitly designed to reproduce multiple key events of the skin sensitization AOP.

## 4. Beyond Structural Alerts and QSAR: Molecular Simulations as Mechanistic Layers for Artificial Intelligence

Within this context, where conventional computational models often fall short of explicitly representing biological mechanisms, molecular docking and molecular dynamics (MD) simulations offer a promising opportunity to incorporate mechanistic information into computational models for skin sensitization. Molecular docking enables the three-dimensional modeling of interactions between a small molecule and a target protein, providing insights into binding modes, molecular orientation, proximity to functional residues, and specific interaction patterns. Beyond estimating binding affinity, docking simulations generate a wide range of quantitative descriptors, including hydrogen-bond networks, hydrophobic contacts, electrostatic interactions, binding energies, and interactions with potentially reactive amino acid residues [25,26]. Such information is particularly relevant for skin sensitization, where the molecular initiating event is driven by the interaction of electrophilic chemicals with nucleophilic residues in skin proteins [27].

Molecular dynamics simulations extend this approach by incorporating the temporal dimension of biomolecular interactions. Whereas docking provides a static representation of a potential binding pose, MD simulations allow the evaluation of conformational stability, the persistence of protein–ligand contacts, residue flexibility, and the temporal evolution of molecular interactions under defined temperature, pressure, and pH conditions. These simulations generate dynamic descriptors such as root mean square deviation (RMSD), root mean square fluctuation (RMSF), radius of gyration, contact occupancy frequencies, average distances to functional residues, and energetic parameters associated with complex stability [20]. Collectively, these descriptors provide a richer and more biologically meaningful characterization of molecular behavior than conventional structural or physicochemical representations. Applications of molecular docking and molecular dynamics have already demonstrated their potential in computational toxicology, including studies addressing skin sensitization and related toxicological endpoints [28].

More importantly, the greatest value of these methodologies lies not only in generating mechanistic hypotheses but also in producing novel descriptor layers that can be incorporated into artificial intelligence models. In recent years, increasing attention has been devoted to the use of interaction fingerprints, three-dimensional structural descriptors, and simulation-derived molecular features as inputs for machine learning algorithms [26,29]. These representations complement traditional chemical fingerprints by incorporating information related to molecular recognition, protein–ligand interactions, and the dynamic stability of biological complexes. Recent studies have shown that interaction-based fingerprints can improve both model interpretability and the identification of structure–activity relationships that remain inaccessible using conventional chemical descriptors alone.

From the perspective of computational toxicology, this strategy is particularly attractive because it enables computational models to more closely reflect the biological events described within the skin sensitization AOP. While conventional QSAR models primarily address the question of which structural features are associated with sensitization, descriptors derived from molecular docking and molecular dynamics provide additional insight into how, where, and with what degree of stability a molecule may interact with biologically relevant proteins. This distinction is especially important because the quality of molecular representations is one of the major determinants of machine learning performance. Consequently, incorporating descriptors derived from biomolecular interactions has the potential to enrich traditional chemical representations with biologically meaningful information, thereby providing artificial intelligence algorithms with features that more closely reflect the underlying mechanisms of toxicity, as demonstrated by Lai et al. and Gu et al. [21,22,28].

The availability of mechanistically informed descriptors generated through molecular docking and molecular dynamics, together with descriptors obtained from other computational methodologies, opens the possibility of developing deep learning architectures—including recurrent neural networks (RNNs), multilayer perceptrons (MLPs), and other deep neural network models—that can learn complex relationships across multiple biological events involved in the skin sensitization AOP [30,31]. Unlike conventional QSAR models, which generally rely on predefined sets of physicochemical or structural descriptors, deep learning approaches can simultaneously integrate heterogeneous sources of information, identifying nonlinear relationships that are difficult to capture using traditional machine learning algorithms. Within this framework, descriptors derived from protein–ligand interactions, molecular dynamics trajectories, and energetic profiles may function as computational representations of key biological events associated with skin sensitization. The convergence of molecular simulation and artificial intelligence therefore represents a promising opportunity to develop a new generation of predictive models that combine high predictive performance with enhanced mechanistic relevance within a unified computational framework.

The generation of these mechanistically informed fingerprints also provides a more explicit representation of biological processes associated with the AOP, particularly those related to the molecular initiating event and the early interactions between electrophilic compounds and skin proteins [7]. As a result, future predictive models may benefit from molecular representations that more closely resemble the biological mechanisms underlying the toxicological endpoint, thereby facilitating the biological interpretation of model predictions and providing a stronger mechanistic rationale for understanding how specific molecular characteristics contribute to the progression of skin sensitization.

Nevertheless, incorporating molecular simulations into computational toxicology also presents important challenges. The quality of the descriptors generated depends heavily on the availability of reliable protein structures, the accuracy of scoring functions, the adequate representation of molecular flexibility, and the reproducibility of simulation protocols. Furthermore, the computational cost associated with molecular dynamics simulations may limit their large-scale application to extensive chemical libraries. Finally, although these methodologies provide valuable structural and biophysical information, they remain unable to fully capture complex biological processes such as metabolic activation, cellular regulation, or adaptive immune responses. Consequently, they cannot comprehensively represent every key event within the skin sensitization AOP.

For these reasons, their greatest value may lie in serving as a complementary mechanistic enrichment layer within future hybrid artificial intelligence models and integrated computational toxicology frameworks.

Within this perspective, integrating molecular simulations—including molecular docking and molecular dynamics—into early-stage cosmetic formulation screening offers an opportunity to enrich the information available during ingredient prioritization by generating descriptor layers that capture mechanistic features not represented in conventional computational models. This concept supports the development of an integrated computational workflow that combines molecular docking, molecular dynamics, mechanistically informed descriptor generation, and machine learning algorithms to facilitate evidence-based decision-making throughout rapid cosmetic innovation cycles.

## 5. Framework-Development Strategy and Evidence-to-Design Traceability

The proposed framework was developed through a structured conceptual-synthesis process comprising five stages: biological and regulatory anchoring, mapping of existing computational approaches, operational identification of methodological gaps, selection of candidate computational components, and integration into a traceable architecture. The objective was not to quantitatively rank published models, but to explain how the available evidence was translated into the proposed design. Accordingly, the analysis considered not only algorithms, but also molecular representations, biological inputs, AOP coverage, validation procedures, applicability domains, uncertainty strategies, and intended contexts of use [15,16,17,18,19,24,32,33].

To make this translation process explicit, Table 2 summarizes the evidence categories considered, the principal limitations or needs identified for each category, the corresponding framework-design requirements, the computational components selected in response, and the proposed benchmarks for their future validation. The table therefore provides the traceability structure linking the review of the existing landscape with the design of the proposed framework.

### 5.1. Biological and Regulatory Anchoring

The skin sensitization AOP was adopted as the principal biological scaffold because it links covalent protein modification with keratinocyte activation, dendritic-cell responses, T-cell activation, and allergic contact dermatitis [7]. OECD-recognized NAMs were mapped to their corresponding biological events: DPRA and kDPRA were considered experimental references for the molecular initiating event, NRF2/ARE-based assays such as KeratinoSens were considered references for keratinocyte activation, and h-CLAT and related assays were considered references for dendritic-cell activation [8,9,10,11].

This mapping also defined the biological scale of the framework. The MIE and selected molecular processes associated with KE2 were prioritized because chemical reactivity, residue-level interactions, molecular recognition, and conformational behavior can be formulated as computationally testable hypotheses [20,25,26,27,35,36,37,38]. By contrast, KE3 and KE4 were not incorporated as explicit simulation modules because antigen processing, dendritic-cell migration, T-cell priming, clonal expansion, and immune memory involve cellular and multicellular processes that cannot be adequately represented through molecular docking or conventional molecular dynamics [7,18,19,20,24]. Their exclusion therefore reflects the intended molecular scale of the framework rather than a lower biological relevance.

### 5.2. Mapping of Existing Computational Approaches

Existing computational approaches were grouped into structural-alert systems, analogue-based and read-across methods, conventional QSAR and machine learning models, AOP-informed or multitask artificial intelligence models, and integrated or Defined Approaches [11,12,13,15,16,17,18,19,23,24,34]. For each category, the analysis considered the prediction target, molecular or biological inputs, molecular representation, AOP coverage, predicted output, validation strategy, applicability-domain procedure, uncertainty treatment, interpretability, and regulatory status [15,16,17,18,19,24].

Because published models differ substantially in their datasets, reference standards, endpoints, and validation procedures, reported predictive metrics were not treated as directly comparable. Instead, the analysis focused on the type of information represented, the biological event addressed, the degree of mechanistic traceability, and the procedures used to define prediction reliability.

### 5.3. Operational Identification of Gaps

Methodological gaps were identified using five predefined dimensions. A representation gap was assigned when models relied mainly on structural alerts, 2D fingerprints, physicochemical properties, molecular graphs, or assay-derived labels without generating reactive-geometry, residue-level, or time-dependent interaction descriptors [12,15,26,30,31,32,33]. Biological-coverage and mechanistic-traceability gaps were identified when AOP events were predicted statistically without explicitly representing the molecular processes involved or linking predictions to defined mechanisms, residues, interactions, or conformational responses [20,24]. Validation gaps included insufficient external evaluation, applicability-domain analysis, uncertainty estimation, or treatment of inconclusive predictions, whereas translation gaps reflected the absence of a clearly defined scientific, regulatory, or industrial decision context [15,16,17]. These analytical dimensions were subsequently applied to the evidence categories summarized in Table 2, allowing each identified limitation to be translated into an explicit framework-design requirement.

The evidence map showed that existing approaches provide substantial and complementary value through analogue inference, statistical structure–activity relationships, assay-outcome prediction, and formal evidence integration [15,16,17,18,19,23,24]. The remaining opportunity was therefore not defined as an absence of predictive models or AOP-informed methods. Instead, it was defined more specifically as the limited availability of intermediate molecular representations capable of describing reactive orientation, residue accessibility, interaction persistence, and conformational behavior before model training [20,21,22,25,26,27,29,30,31,32]. This distinction informed the central objective of the framework: to determine whether simulation-derived descriptors provide information that is complementary to conventional chemical representations and existing key-event predictions.

### 5.4. Selection of Candidate Computational Components

As summarized in Table 2, each identified limitation or need was translated into a specific design requirement and linked to a candidate computational response and validation benchmark. Candidate components were then evaluated according to biological anchoring, potential nonredundancy with conventional descriptors, mechanistic traceability, availability of an experimental benchmark, technical feasibility, modularity, scalability, uncertainty assessment, and compatibility with existing NAMs [7,8,9,10,11,15,16,17,18,19,20]. Components were retained only when a plausible biological rationale, a defined computational output, a principal assumption, and a potential validation benchmark could be specified.

Structural alerts and reaction-domain information were retained as the initial layer because they capture established knowledge on electrophilic mechanisms and chemical-activation pathways [12,13,23,36]. Covalent docking was selected to assess the geometric feasibility, orientation, and accessibility of predefined electrophile–nucleophile interaction hypotheses [25,27,35]. Molecular dynamics was incorporated to introduce a temporal layer capable of characterizing contact persistence, residue flexibility, solvent exposure, and conformational stability [20,21]. These methods are not proposed as direct substitutes for experimental peptide-reactivity assays; rather, they are intended as sources of descriptors associated with selected physicochemical determinants of peptide reactivity and adduct behavior [8,20,21,26,27,35,36].

A KEAP1-centered module was included as a molecular proxy for selected KE2-related processes because perturbation of the KEAP1–NRF2 regulatory axis provides a plausible mechanistic link with NRF2/ARE-based keratinocyte assays. However, this module is not intended to reproduce keratinocyte activation in full. Its role is to generate molecular descriptors associated with explicitly defined covalent or non-covalent KEAP1 interaction hypotheses, which should subsequently be evaluated against KeratinoSens or related NRF2/ARE data [9,37,38].

Conventional structural, physicochemical, reactivity, and toxicokinetic descriptors were retained because the purpose of the framework is not to replace established molecular representations, but to determine whether simulation-derived features provide incremental information. A multimodal machine learning layer was therefore proposed to integrate complementary descriptor blocks while preserving the ability to examine their individual contributions through ablation and feature attribution analyses. The framework does not prescribe a specific algorithm; simpler statistical and machine learning baselines should be evaluated alongside deep learning architectures to determine whether additional model complexity is justified by the available data [15,21,29,30,31,32,33,34].

### 5.5. Framework Integration and Intended Decision Context

The selected components were integrated into a modular architecture linking each evidence source and identified limitation to a design requirement, computational response, and validation benchmark, as summarized in Table 2. The workflow comprises: (i) a conventional chemical-information layer; (ii) a reaction-domain and MIE/KE1 descriptor layer; (iii) a KE2 molecular-proxy layer; (iv) a multimodal integration layer; and (v) an applicability-domain, uncertainty, and decision-support layer. This modular organization enables each descriptor block to be evaluated independently before integration into a final predictive model.

The initial intended context of use was defined as early hazard prioritization of structurally characterized cosmetic ingredients. The framework is intended to identify substances requiring further evaluation and to provide a mechanistic evidence profile supporting the prioritization of experimental NAMs. It is not presented as a stand-alone method for regulatory classification, potency categorization, consumer risk assessment, or exposure-based safety determination [16,19,24]. This distinction defines the intended output of the framework and prevents the proposed architecture from being interpreted as a validated DA.

Framework development was conducted through iterative discussion among the authors rather than through a formal Delphi or external consensus process. To improve transparency, each proposed component was linked to its supporting evidence, identified gap, design requirement, principal assumption, and potential experimental benchmark. The absence of formal external consensus is acknowledged as a limitation of the framework-development process.

This methodological process was designed to support conceptual framework development and should not be interpreted as a meta-analysis of predictive performance or as formal regulatory validation. Considerable heterogeneity exists across available models with respect to prediction targets, datasets, reference standards, molecular representations, and validation strategies, preventing direct quantitative comparison of reported performance metrics [15,16,17,18,19,24]. Furthermore, part of the evidence supporting docking, molecular dynamics, and simulation-derived descriptors originates from adjacent fields such as structural bioinformatics and drug discovery and therefore represents methodological transfer rather than direct validation for skin sensitization [21,22,26,29,30,31,32,33,35]. The resulting architecture should consequently be interpreted as a transparent and testable conceptual framework requiring module-level, external, and prospective validation.

## 6. Next-Generation Computational Approaches for Skin Sensitization: Bridging AOP Biology, Molecular Modeling and Artificial Intelligence

Building upon the remarkable progress achieved by computational methodologies as evidence-support tools for early decision-making in skin sensitization assessment during cosmetic product development, we propose an integrated computational workflow structured around the AOP framework. The objective of this workflow is to predict the skin sensitization potential of chemicals through mechanistically informed molecular descriptors that reproduce key biological events involved in the sensitization process. Specifically, the proposed framework focuses on developing computational counterparts for Molecular Initial Event (MIE), corresponding to the covalent interaction of electrophilic chemicals with skin proteins, and KE2, representing keratinocyte activation through perturbation of the KEAP1–NRF2–ARE signaling pathway.

To reproduce MIE computationally, the proposed strategy takes the DPRA as its biological reference. Experimentally, DPRA quantifies the depletion of synthetic peptides containing nucleophilic lysine and cysteine residues following their reaction with electrophilic compounds. Accordingly, its computational counterpart consists of an automated workflow integrating covalent molecular docking [35] and molecular dynamics simulations using a panel of peptides derived from those employed in the experimental assay [23,36]. This framework enables the generation of mechanistically relevant descriptors describing peptide–ligand interactions, including interaction patterns with key nucleophilic residues such as Lys and Cys, residue accessibility, binding energies, and the geometric orientation of the electrophilic group relative to the reactive amino acids. These descriptors are subsequently incorporated into deep learning models to support predictive model training.

Although this computational representation of MIE captures important molecular features associated with peptide reactivity, it cannot fully reproduce the biological complexity of the haptenation process. Several biologically relevant phenomena—including oxidation reactions, metabolic bioactivation, microenvironmental effects, and other intrinsic biological processes involved in chemical activation—remain outside the scope of current molecular simulation approaches [36]. Nevertheless, rather than attempting to replicate every biological process involved in haptenation, the proposed workflow seeks to generate an additional layer of mechanistically relevant descriptors that complement existing computational evidence. This strategy may be particularly valuable for compounds behaving as prehaptens or prohaptens, whose sensitization potential frequently depends on oxidative or metabolic activation and is currently addressed through mechanistic alerts incorporated into platforms such as the OECD QSAR Toolbox [13].

The computational counterpart of KE2 is subsequently constructed by evaluating each candidate molecule against biologically relevant domains of KEAP1. The rationale behind this module is that perturbation of the KEAP1–NRF2 signaling axis constitutes one of the earliest cellular responses to electrophilic stress and represents the mechanistic basis of the KeratinoSens assay. Like the MIE workflow, this module integrates molecular docking and molecular dynamics simulations to generate descriptors associated with protein–ligand recognition. These include binding topologies, interactions with functionally important amino acid residues, local conformational changes, predicted binding affinities, interaction profiles, and the formation of non-covalent contacts, with particular emphasis on cysteine residues Cys151, Cys273, and Cys288, which have been proposed as primary electrophilic sensors within KEAP1 [37,38]. Importantly, the objective of this computational representation is not to simulate keratinocyte activation itself, but rather to characterize the ability of a chemical to perturb the KEAP1–NRF2 regulatory axis, thereby generating molecular descriptors associated with one of the central biological mechanisms governing the antioxidant response preceding keratinocyte activation.

The final stage of the workflow consists of integrating descriptors generated from the computational representations of MIE and KE2 with conventional molecular descriptors already employed in regulatory toxicology, including physicochemical properties, structural descriptors, chemical alerts, reactivity profiles, and simple toxicokinetic parameters available through established OECD computational frameworks. The resulting multidimensional molecular representation is intended to provide a more comprehensive mechanistic basis for predicting the skin sensitization potential of novel compounds.

To exploit this enriched descriptor space, the workflow proposes the implementation of advanced machine learning or deep learning architectures, such as multitask QSAR models similar to those described by Zhang et al. [34]. Within these architectures, each molecule is encoded through multiple complementary descriptor sets, allowing the model to integrate structural, physicochemical, mechanistic, and biological information simultaneously before classifying compounds according to their skin sensitization potential. Similar descriptor-encoding strategies have recently gained considerable attention in cheminformatics, where modern molecular representations have progressively evolved from handcrafted descriptors toward learned latent embeddings capable of capturing increasingly complex biological information [32,33].

**Figure 1:**
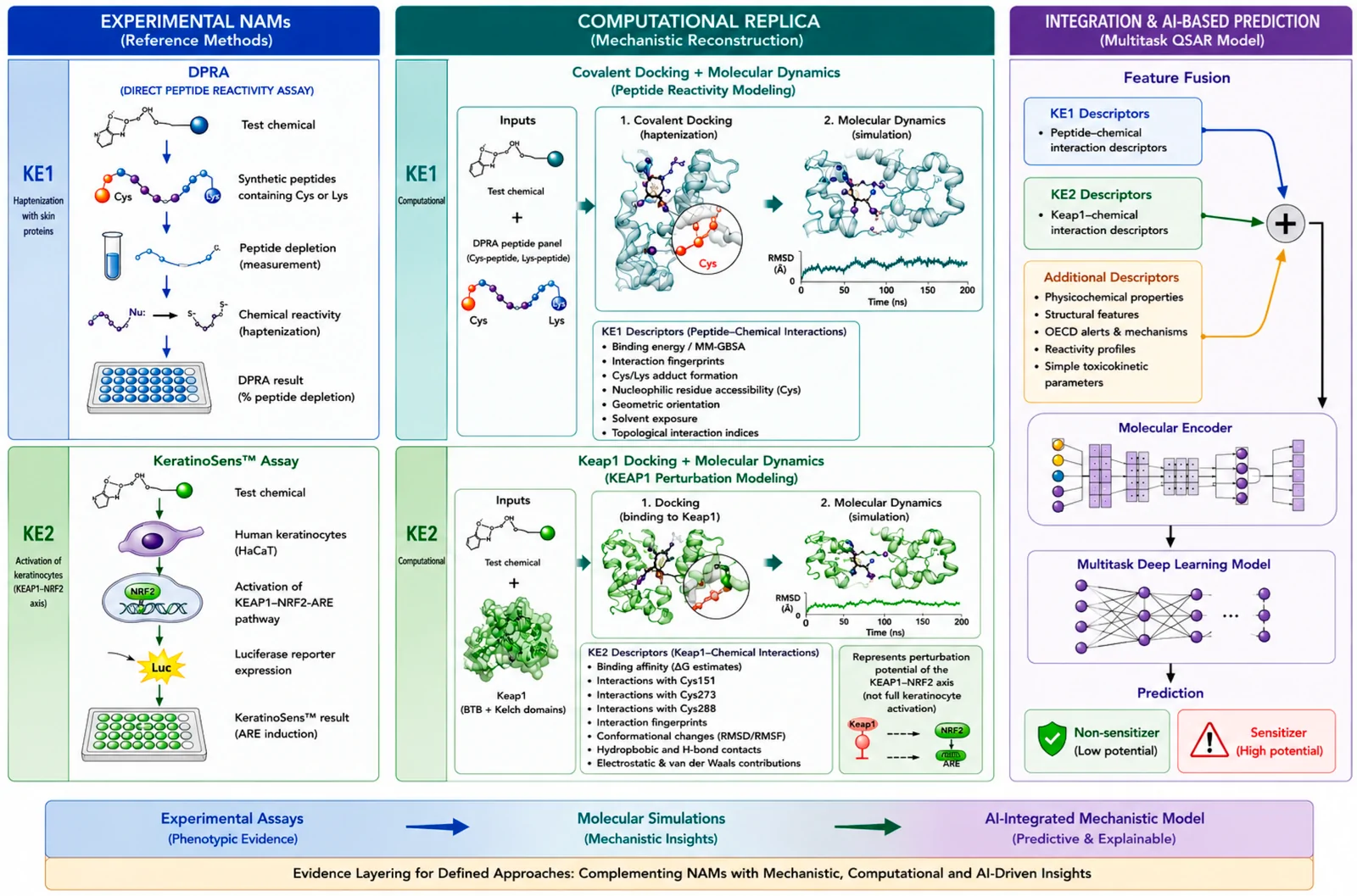
Conceptual workflow comparing experimental NAMs and their computational counterparts for the first two key events of the skin sensitization Adverse Outcome Pathway. The left panel summarizes the reference experimental methods: the Direct Peptide Reactivity Assay (DPRA) for KE1, corresponding to haptenization of skin proteins, and the KeratinoSens™ assay. The central panel presents the proposed computational reconstruction of both key events. The right panel illustrates the integration of KE1- and KE2-derived descriptors with physicochemical properties, structural features, OECD-derived mechanistic information, chemical reactivity profiles, and simple toxicokinetic parameters. These complementary features are encoded into a multitask deep learning model to predict skin sensitization potential. The figure was generated using ChatGPT 5.5 (OpenAI; https://chatgpt.com) as an illustration support tool. The scientific concept, content, layout, and final design were conceived, specified, reviewed, and approved by the authors. Accessed on 22 July 2026.

From this perspective, the proposed workflow establishes the conceptual foundation for the future development of a conceptual computational framework capable of providing an additional layer of evidence during early decision-making in cosmetic innovation, as shown in Figure 1. Rather than replacing experimental NAMs, this framework is envisioned as a complementary strategy that supports the prioritization of compounds requiring further experimental evaluation, thereby improving the efficiency of laboratory testing while reducing both development time and associated costs.

Overall, the proposed computational workflow represents a conceptual evolution beyond traditional QSAR methodologies by explicitly incorporating mechanistic information derived from key biological events within the skin sensitization AOP. Integrating molecular docking, molecular dynamics simulations, MIE- and KE2-derived interaction descriptors, and conventional structural and physicochemical information provides an opportunity to develop predictive models that are not only more accurate but also biologically interpretable. Although important challenges remain—including the accurate representation of covalent chemistry, the availability of high-quality experimental data, computational scalability, and eventual regulatory acceptance—this framework outlines a promising direction toward the development of next-generation computational frameworks.

Rather than replacing existing experimental methodologies, the proposed strategy seeks to complement the evidence generated by current NAMs, providing an additional computational layer to support early ingredient prioritization, reduce development costs, and accelerate innovation within the cosmetics industry. In this context, the convergence of mechanistic biology, molecular modeling, and artificial intelligence has the potential to become one of the fundamental pillars of the next generation of evidence-based cosmetic safety assessment.

Finally, from an industrial perspective, the greatest value of this framework lies in its ability to support decision-making during the earliest stages of cosmetic product development, including the selection of active ingredients, excipients, and formulation stabilizers. Early identification of ingredients with sensitization potential, together with the rational prioritization of safer alternatives and a more strategic implementation of NAMs, may substantially reduce development timelines, minimize late-stage reformulations, and improve the overall efficiency of innovation programs. As the cosmetics industry continues to respond to rapidly evolving consumer demands without compromising product safety, the convergence of mechanistic biology, molecular simulations, and artificial intelligence is poised to become a cornerstone of future evidence-based safety assessment strategies. Rather than replacing experimental testing, these computational approaches have the potential to make it more informed, targeted, and efficient, ultimately facilitating the development of innovative cosmetic products with greater scientific confidence and regulatory credibility.

## 7. Applicability Domain Definition and Future Validation Strategy

The initial chemical domain is expected to include structurally defined, small organic molecules with assignable protonation states and sufficiently characterized electrophilic mechanisms [16,17]. Complex mixtures, botanical extracts, polymers, metals, nanomaterials, highly insoluble materials, and chemicals with poorly characterized transformation products should initially be considered outside the domain. Predictions for substances requiring oxidative or metabolic activation should be interpreted with caution unless the relevant products are explicitly generated and evaluated [13,23,36].

Validation should proceed sequentially rather than evaluating only the final integrated model. The MIE module should first be benchmarked against DPRA peptide depletion and, where available, kinetic peptide-reactivity data [8,36]. The KE2 molecular-proxy module should be evaluated against KeratinoSens or related NRF2/ARE outcomes, while acknowledging that correspondence between a molecular proxy and a cellular response may be incomplete [9,37,38]. Only after demonstrating reproducible associations at the module level should the descriptor blocks be integrated into a final predictive model [11,24].

Model development should use chemically informed data partitioning, including scaffold-based or temporal splits, to reduce information leakage between closely related analogues. Final evaluation should be performed using an external dataset that was not used for descriptor selection, model optimization, or hyperparameter tuning [15,16,17]. Predictions should also include calibrated uncertainty estimates and allow an inconclusive or out-of-domain outcome rather than forcing every chemical into a positive or negative category [11,15,16,17,24].

Simulation-derived descriptors should also be evaluated for technical reproducibility. This assessment should include independent trajectories, alternative initial conformations, force-field sensitivity, stability of descriptor values, and reproducibility across software implementations where feasible [20,21,25,35]. A descriptor that varies substantially between technically valid simulations may not be suitable as a machine learning input, regardless of its apparent biological interpretation [15].

Claims concerning reduced development time, lower cost, or prevention of late-stage reformulation should remain conditional until prospectively evaluated. Relevant implementation metrics would include computational time per chemical, experimental tests avoided, inconclusive predictions, false-negative rates, prioritization accuracy, time to decision, and prospective concordance with NAM outcomes.

## 8. Conclusions

The principal contribution of this perspective is not the presentation of a validated skin sensitization model, but the formulation of a transparent and testable architecture for incorporating physics-informed molecular descriptors into AOP-guided prediction. Existing expert systems, QSAR models, AOP-informed artificial intelligence approaches, and regulatory-defined approaches provide essential benchmarks and should not be replaced. Instead, the proposed framework is intended to investigate whether covalent geometry, residue-level interactions, and conformational dynamics provide complementary information for early ingredient prioritization. From an industrial perspective, it is conceived as an actionable framework that can be implemented progressively, according to the successive layers of information generated, in order to support informed decision-making, enable the early identification of potential sensitization risks, and guide the prioritization of candidates requiring additional experimental evaluation. Its scientific, industrial, and regulatory value will ultimately depend on module-level validation, demonstration of incremental predictive performance, explicit uncertainty characterization, computational scalability, and prospective comparison with established NAMs and defined approaches.

## Figures and Tables

**Table 1:** Scientific capabilities comparison of representative computational approaches for skin sensitization assessment and the distinctive contribution of the proposed framework, where ✗ indicates a lack of evidence supporting the capability and ✓ indicates that evidence exists but without a specific level [12,13,14,15,16,17,18,19,20,21,22,23].

Feature	Structural Alerts	Read-Across	QSAR	ML	Defined Approaches	Proposed Framework
Mechanistic interpretability	High	Moderate	Low	Low	High	High
Protein structural information	✗	✗	✗	✗	✗	✓
Time-dependent information	✗	✗	✗	✗	✗	✓
Residue-level interactions	✗	✗	✗	✗	✗	✓
Physics-based descriptors	✗	✗	✗	✗	✗	✓
AOP-guided	Partial	Partial	Partial	Variable	✓	✓
Experimental evidence required	No	Existing analogues	Existing datasets	Existing datasets	Yes	No (conceptual computational layer)
Applicability domain	Moderate	Narrow	Moderate	Variable	High	To be established
Regulatory acceptance	High	High	Moderate	Low	High	Future validation required

**Table 2:** Evidence-to-design traceability matrix for the proposed computational framework.

Evidence Category	Limitation or Need Identified	Framework Design Requirement	Selected Component	Rationale for Inclusion	Proposed Validation Benchmark
Industrial ingredient-prioritization context	Full molecular simulation may not be feasible for every candidate, and hazard prediction alone does not constitute risk assessment [20,21,24]	Define a tiered context of use and restrict high-cost calculations to priority cases	Tiered screening and prioritization strategy	Aligns computational depth with decision value and available evidence	Prospective cosmetic case studies, time-to-decision, experimental tests prioritized, and false-negative analysis
Skin sensitization AOP and OECD NAMs [7,8,9,10,11]	No individual information source represents the complete biological process [7,11,18,19]	Anchor each computational module to a defined biological or experimental reference	MIE and KE2-related descriptor modules	Provides biological organization and avoids selection of computational targets without a defined toxicological rationale	DPRA/kDPRA for MIE; KeratinoSens or related NRF2/ARE data for KE2-related processes
Structural alerts and read-across [12,13]	Alerts may overpredict, while analogue inference can be affected by activity cliffs and limited analogue availability [12,13,14]	Add molecule-specific information on reaction mechanism, accessibility, and interaction geometry	Reaction-domain descriptors and covalent-docking module	Complements substructure and analogue information with explicit reactive hypotheses	DPRA/kDPRA outcomes, reaction-domain classification, and matched molecular-pair analysis
Conventional QSAR and machine learning models [15,16,17,18,19,24]	Predictions rely predominantly on statistical associations and may be unreliable outside the training domain [15,16,17,24]	Generate orthogonal descriptors and retain explicit applicability-domain assessment	Conventional descriptors combined with simulation-derived descriptor blocks	Tests whether interaction-level information adds value beyond two-dimensional representations	External scaffold-based or temporal validation; comparison with conventional QSAR baselines
AOP-informed and multitask models	Key events may be predicted from molecular structure or assay labels without explicit molecular interaction descriptors [24,30,31,34]	Introduce molecular proxies upstream of key-event prediction	Separate MIE and KE2-related descriptor blocks	Increases traceability between molecular features and the biological event being represented	Individual KE datasets followed by integrated adverse-outcome evaluation
Molecular docking [25,26,27,35]	Provides primarily static poses and is sensitive to target preparation, reactive-site assignment, and scoring assumptions [25,27,35]	Add temporal assessment and predefined quality-control criteria	Covalent or mechanism-specific docking followed by selected MD simulations	Characterizes reactive orientation and provides initial structures for dynamic assessment	Pose reproducibility, reactive-geometry criteria, peptide-reactivity data, and comparison across docking protocols
Molecular dynamics [20,21]	High computational cost, sampling uncertainty, and sensitivity to force fields and initial conditions [20,21]	Use MD selectively and evaluate descriptor reproducibility	Tiered MD module with replicate trajectories and convergence assessment	Generates contact-persistence, flexibility, solvent-exposure, and conformational descriptors	Independent trajectory replicates, sensitivity analyses, descriptor stability, and experimental KE associations
Interaction fingerprints and multimodal representations [21,22,26,29,30,31,32,33,34]	Heterogeneous information sources are frequently modeled separately, and added complexity may not provide incremental value [21,22,26,29,30,31,32,33,34]	Integrate descriptor blocks while preserving the ability to evaluate each block independently	Multimodal machine learning architecture with ablation analysis	Allows structural, physicochemical, reactivity, docking, and MD information to be combined and tested	Descriptor-block ablation, external validation, calibration, and stable feature attribution

## Data Availability

The raw data supporting the conclusions of this article will be made available by the authors on request.

## Abbreviations

The following abbreviations are used in this manuscript:

AOP	Adverse Outcome Pathway
Cys	Cysteine
DA	Defined Approach
DAs	Defined Approaches
DPRA	Direct Peptide Reactivity Assay
h-CLAT	Human Cell Line Activation Test
IL-8 Luc	Interleukin-8 Luciferase Assay
KEs	Key Events
KE2	Key Event 2
KEAP1	Kelch-like ECH-Associated Protein 1
Lys	Lysine
MIE	Molecular Initial Event
MLPs	Multilayer Perceptrons
MD	Molecular Dynamics
NAMs	New Approach Methodologies
NRF2	Nuclear Factor Erythroid 2-Related Factor 2
OECD	Organisation for Economic Co-operation and Development
QSAR	Quantitative Structure–Activity Relationship
RMSD	Root Mean Square Deviation
RMSF	Root Mean Square Fluctuation
RNNs	Recurrent Neural Networks
U-SENS	U-SENS™ Assay
